# Aging and amyloid β oligomers enhance TLR4 expression, LPS-induced Ca^2+^ responses, and neuron cell death in cultured rat hippocampal neurons

**DOI:** 10.1186/s12974-017-0802-0

**Published:** 2017-01-31

**Authors:** María Calvo-Rodríguez, Carmen de la Fuente, Mónica García-Durillo, Carmen García-Rodríguez, Carlos Villalobos, Lucía Núñez

**Affiliations:** 1 Instituto de Biología y Genética Molecular (IBGM), Universidad de Valladolid y Consejo Superior de Investigaciones Científicas (CSIC), Valladolid, Spain; 2 Departamento de Bioquímica y Biología Molecular y Fisiología, Universidad de Valladolid, Valladolid, Spain

**Keywords:** Aging, TLR4, Alzheimer’s disease, Hippocampal neurons, Calcium, Amyloid β oligomers

## Abstract

**Background:**

Toll-like receptors (TLRs) are transmembrane pattern-recognition receptors of the innate immune system recognizing diverse pathogen-derived and tissue damage-related ligands. It has been suggested that TLR signaling contributes to the pathogenesis of age-related, neurodegenerative diseases, including Alzheimer’s disease (AD). AD is associated to oligomers of the amyloid β peptide (Aβo) that cause intracellular Ca^2+^ dishomeostasis and neuron cell death in rat hippocampal neurons. Here we assessed the interplay between inflammation and Aβo in long-term cultures of rat hippocampal neurons, an in vitro model of neuron aging and/or senescence.

**Methods:**

Ca^2+^ imaging and immunofluorescence against annexin V and TLR4 were applied in short- and long-term cultures of rat hippocampal neurons to test the effects of TLR4-agonist LPS and Aβo on cytosolic [Ca^2+^] and on apoptosis as well as on expression of TLR4.

**Results:**

LPS increases cytosolic [Ca^2+^] and promotes apoptosis in rat hippocampal neurons in long-term culture considered aged and/or senescent neurons, but not in short-term cultured neurons considered young neurons. TLR4 antagonist CAY10614 prevents both effects. TLR4 expression in rat hippocampal neurons is significantly larger in aged hippocampal cultures. Treatment of aged hippocampal cultures with Aβo increases TLR4 expression and enhances LPS-induced Ca^2+^ responses and neuron cell death.

**Conclusions:**

Aging and amyloid β oligomers, the neurotoxin involved in Alzheimer’s disease, enhance TLR4 expression as well as LPS-induced Ca^2+^ responses and neuron cell death in rat hippocampal neurons aged in vitro.

**Electronic supplementary material:**

The online version of this article (doi:10.1186/s12974-017-0802-0) contains supplementary material, which is available to authorized users.

## Background

Toll-like receptors (TLRs) are a family of innate immune system receptors involved in sensing and response to pathogen-associated molecular patterns (PAMPs) and endogenous ligands known as damage-associated molecular patterns (DAMPs) that are released upon cell damage and necrosis [1]. Toll proteins were discovered in *Drosophila melanogaster* [2]. Later, a mammalian homologue for Toll was found, and TLR4, the receptor for lipopolysaccharide (LPS) present in Gram-negative bacteria, was identified [3, 4]. TLRs are widely expressed in a diversity of mammalian immune and non-immune cells, and they are present in the brain, where their expression is not restricted to microglia [5] but expands to astrocytes [6], oligodendrocytes [7], and neurons [8]. The functional implication of TLR expression in neurons is not well understood yet. It has been proposed that TLR4 may contribute to neural plasticity and development in neurons [9]. In addition, recent studies indicate that TLR4 expression is upregulated with normal aging [10], suggesting an altered regulation of the innate immune response in aging that may be relevant in different neurodegenerative disorders such as Alzheimer’s disease (AD). AD, the most common form of dementia, is strongly associated to aging and is characterized by the gradual loss of memory and cognitive function. The causes for AD are still not well understood. However, it is well known that AD is associated to formation of amyloid plaques made up of amyloid β peptide (Aβ), mainly Aβ_1–42_, derived from the altered metabolism of the amyloid precursor protein after being processed by β- and γ-secretases. AD is also linked to intracellular neurofibrillary tangles, composed of abnormally hyper phosphorylated tau protein [11].

Interestingly, McGeer and McGeer proposed in the late 1980s that innate immunity had an important role in neurodegenerative diseases (revised in [11, 12]). Although this theory was not well accepted initially, consensus is growing about the involvement of an inflammatory component in AD. Microglia is responsible for immunity in the brain and becomes activated by signals released by surrounding cells. In the AD brain, the sites of neuroinflammation are surrounding senile plaques, which show increased levels of pro-inflammatory factors, such as pro-inflammatory cytokines, complement components, and proteases [12, 13]. Recently, the *tlr4* gene has emerged as a candidate susceptibility gene for AD. For example, a genetic study proposed that a polymorphism in TLR4 (Asp299Gly) may decrease the risk of AD independently of a polymorphism in apolipoprotein E, suggesting the involvement of the innate immunity in neurodegeneration in general, and of TLR4 in AD, in particular [14, 15]. Consistently, AD brains show increased expression of TLR4 [15]. Furthermore, this receptor plays an important role in microglial neurotoxicity, since LPS binding induces its activation, thus releasing toxic substances to neurons [16]. Consequently, instead of counteracting the damage caused by pathogens, TLR4 activation may lead to increased damage due to the release of toxic factors such as nitric oxide and oxygen free radicals [17]. In this manner, it appears that Aβ may sensitize microglia to stimulation by some TLR ligands like LPS [18] since co-administration of Aβ and LPS increases activation of TLR4, leading to increased release of nitric oxide and tumor necrosis factor α [19]. However, the possible interactions of LPS and Aβ on hippocampal neurons have not been assessed yet. In this work, we aimed at investigating the interplay between neuroinflammation and AD in the context of aging.

To accomplish the above goal, we have employed here aged cultures of rat hippocampal neurons that are considered a model of aging and/or senescence, since some of the changes occurring in the elderly in vivo are mimicked in neurons aged in vitro [20, 21]. Our results show that rat hippocampal neurons express TLR4 and expression increases with time in culture consistently with in vivo aging. We also found that LPS increases cytosolic [Ca^2+^] and promotes neuron cell death only in aged cultures. Treatment with AD-related oligomers of the amyloid β peptide (Aβo) further enhanced TLR4 expression, Ca^2+^ responses induced by LPS and neuron cell death, suggesting the interplay between TLR4 and Aβo in neuron cell death associated to aging and AD.

## Methods

### Animals and reagents

Wistar rat pups were obtained from the Valladolid University animal facility. Fura2/AM was from Invitrogen (Carlsbad, CA). Fetal bovine serum was from Lonza (Basel, Switzerland). Neurobasal medium, Hank’s balanced salt solution, minimal essential medium, B27, l-glutamine, and gentamicin were from Gibco (Carlsbad, CA). Papain solution was from Worthington (Lakewood, NJ). Aβ_1–42_ peptides were purchased from Bachem AG (Bubendorf, Switzerland). TLR4 (H80) antibody was from Santa Cruz Biotechnology (Santa Cruz, CA). Antibody against β III tubulin is from Covance (Princeton, NJ). Antibodies against NR1 (AB9864), NR2A (AB1555P), and NR2B (AB1557P) are from Chemicon International (Millerica, MA). CAY10614 was from Cayman Chem. (Ann Arbor, MI). MK801 is from Sigma (St. Louis, MO). Poly-d-lysine and Annexin V were from BD (Franklin Lakes, NJ). DNase I and LPS from *Escherichia coli* O111B4 were from Sigma (St. Louis, MO). LPS, solved in ultrapure water, was boiled for 1 h to avoid protein contaminants, as reported previously [22]. Other reagents and chemicals were obtained either from Sigma or Merck.

### Primary hippocampal neuron culture

Hippocampal neurons were prepared from P0 Wistar rat pups under sterile conditions as reported by Brewer et al. [23] with further modifications [24]. Briefly, rat pups were sacrificed and, after brain removal, meninges were discarded and hippocampi were separated from the cortex. Hippocampal tissue was cut in small pieces, transferred to papain solution (20 u/ml), and incubated at 37 °C for 30 min with occasional gentle shaking. After 15 min, DNase I (50 μg/ml final concentration) was added. Tissue pieces were washed with Neurobasal medium and cell suspension was obtained using a fire-polished pipette in Neurobasal supplemented with 10% fetal bovine serum. Cells were centrifuged at 160 g for 5 min and pellet was suspended in Neurobasal medium. Hippocampal cells were plated onto poly-d-lysine-coated, 12-mm diameter glass coverslips at 30 × 10^3^ cells/dish, cultured in the same medium supplemented with 2 mM l-glutamine, 1 μg/ml gentamicin, 2% B27, and 10% FBS, and maintained in a humidified 37 °C incubator with 5% CO_2_. Cells were either cultured for 4–8 days in vitro (DIV) for short-term cultures (young neurons), 9–10 DIV for mature neurons, or >18 DIV for long-term cultures (aged and/or senescent neurons) as in previous studies [25, 26].

### Apoptosis

Hippocampal neurons at different DIV were treated with or without 1 μg/ml LPS, 0.25 μM CAY10614, or 1 μM Aβo, and incubated for 48 h at 37 °C/5% CO_2_. Then, cells were washed with phosphate buffered saline once and apoptosis was evaluated using Annexin V (1:20, 10 min) in annexing binding buffer (140 mM NaCl; 2.5 mM CaCl_2_; 10 mM Hepes pH 7.4) and assessed by fluorescence microscopy using a Nikon Eclipse TS100 microscope (objective ×40).

### Immunofluorescence

Hippocampal cells at different days in vitro (DIV) (4–8, 9–10, and >18 DIV) were fixed with 4% p-formaldehyde and incubated with antibodies against TLR4 (1:200) or antibodies against NR1, NR2A, and NR2B (1:100). Immunopositive cells were revealed using an Alexa Fluor 488-tagged antibody (1:300). Optical density was measured in individual neurons using the ImageJ software (National Institute of Mental Health, Bethesda, MA, USA) as reported previously [25]. In some experiments, cells were co-stained with anti-β-tubulin III (1:400) for detection of TLR4 in identified neurons and incubated with 1:100 labeled anti-IgG antibodies as reported previously [25].

### Preparation of Aβ_1–42_ oligomers (Aβo)

Aβo were prepared as reported previously [27]. Briefly, Aβ_1–42_ was initially solved to 1 mM in cold hexafluoroisopropanol and separated into aliquots in sterile microcentrifuge tubes. Hexafluoroisopropanol was removed under vacuum in a Speed Vac concentrator, and the peptide film was stored desiccated at −20 °C. For aggregation, the peptide was first suspended in dry dimethyl sulfoxide to a concentration of 5 mM. For complete resuspension of the peptide, it was subjected to a 10 min of ultrasound, aliquoted in propylene non-siliconized vials, and stored at −20 °C. Minimal essential medium supplemented with 0.5 mg/ml Fe^2+^, 0.5 mg/ml Cu^2+^, and 0.5 mg/ml Zn^2+^ was added to bring the peptide to a concentration of 80 μM, and it was incubated at 37 °C for 24 h. For experiments, Aβo were solved in MEM at a final concentration of 1 μM. Oligomer formation was tested using western blotting as previously reported [27].

### Fluorescence imaging of cytosolic [Ca^2+^]

Cells were incubated in standard external medium (SEM) containing 145 mM NaCl, KCl, 1 mM CaCl_2_, 1 mM MgCl_2_, 10 mM glucose, and 10 mM Hepes 10, pH 7.42), and later loaded with the fura2/AM dye (4 μM) for 60 min at room temperature. Then, coverslips were placed on the perfusion chamber of a Zeiss Axiovert 100 TV, perfused continuously with the same standard medium pre-warmed at 37 °C and epi-illuminated alternately at 340 and 380 nm. Light emitted at 520 nm was recorded every 5 s with a Hamamatsu ER camera (Hamamatsu Photonics France). Perfusion was stopped before adding vehicle or LPS during times indicated in the figures. Pixel by pixel ratios of consecutive frames were captured, and [Ca^2+^]_cyt_ of regions of interest corresponding to individual neurons selected by morphometric characteristics were expressed as the ratio of emission following excitation at 340 and 380 nm (Ratio F340/F380), as reported in detail previously [28]. Analysis of responses in non-neuronal cells was also carried out by selecting cells not showing the morphology of neurons. In addition, the glial phenotype was confirmed by the lack of Ca^2+^ responses to N-methyl d-aspartate (NMDA).

### Statistical analysis

Changes in fluorescence ratio are expressed as Δ[Ca^2+^]_cyt_ (Ratio F340/F380). Calculation of Δ[Ca^2+^]_cyt_ was performed using Origin Lab 7.0. Data are presented as mean ± SEM. When only two means were compared, Student’s *t* test was used. For comparing more than two groups, statistical significance of the data was assessed by one-way ANOVA and compared using Bonferroni’s multiple comparison tests. Differences were considered significant at *p* < 0.05.

## Results

### LPS induces apoptosis only in aged cultures of rat hippocampal neurons

**Fig. 1:**
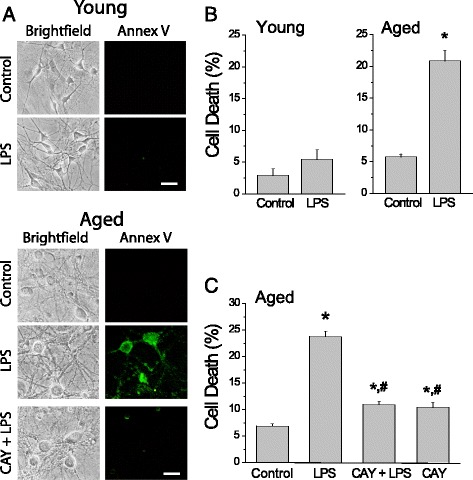
LPS induces cell death in aged cultures of rat hippocampal neurons but not in young cultures. Primary hippocampal neurons cultured for 5–8 and >18 DIV were treated for 48 h in absence (control) or presence of LPS, and apoptosis was assessed 48 h later by staining with Annexin V. **a** Representative bright field and Annexin V inmunofluorescence (Annex V) microphotographs of young (*upper*) and aged (*bottom*) cultured neurons in presence or absence of LPS (1 μg/ml) and CAY10614 (0.25 μM). *Bar* represents 10 μm and applies for all photographs. **b** Percent of neuron dead cells in control and LPS-treated cells. Values represent mean ± SEM of three and nine experiments from independent cultures, respectively. *p < 0.05 compared to control group. **c** Percent of neuron dead cells in control cells and in cells treated with LPS in presence or absence of the antagonist CAY10614. Values represent mean ± SEM of 4 independent experiments from independent cultures. *p < 0.05 compared to control group; ^#^ *p* < 0.05 compared to LPS group

We have investigated whether LPS, a classical TLR4 ligand, induces apoptosis in young (4–8 DIV) and aged (>18 DIV) cultures of rat hippocampal neurons. Young and aged cultures of rat hippocampal neurons were incubated for 48 h with vehicle or 1 μg/ml LPS and then tested for apoptosis by measuring everted phosphatidylserine using Annexin V staining in individual neurons identified by their morphometric characteristics (Fig. 1a, b). Results show that LPS treatment did not induce a statistically significant increase in the fraction (percent) of apoptotic cells in young cultured neurons. However, in aged cultured neurons, LPS enhanced significantly the fraction of apoptotic neurons (Fig. 1a, b). The TLR4-antagonist CAY10614 prevented largely the effect of LPS on aged cultured neurons (Fig. 1c). Therefore, LPS promotes apoptosis only in in vitro aged hippocampal cultures and this effect depends on TLR4.

### Rat hippocampal neurons express TLR4 and its level of expression increases significantly with in vitro aging and/or senescence

**Fig. 2:**
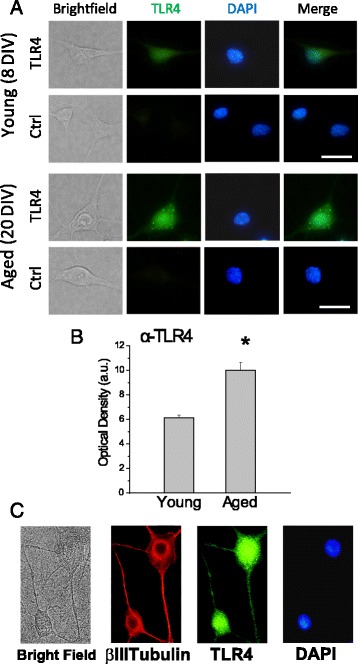
TLR4 is expressed in cultured rat hippocampal neurons and expression levels increases with in vitro aging. **a** Representative immunofluorescence images for TLR4 of young (8 DIV) and aged (20 DIV) hippocampal neurons. Control (Ctrl) refers to images taken from similar cultures in the absence of primary antibody. *Scale bar* is 10 μm and applies to all frames. **b** Quantitative analysis of immunofluorescence intensity levels (Optical Density in arbitrary units) for TLR4. *Bars* represent mean ± SEM (*n* = 40–115 cells from 3–4 independent cultures). *p < 0.05 aged *vs* young. **c** Cells were fixed and tested for immunofluorescence detection of TLR4 in neurons. Pictures correspond to a bright field image, immunostaining of β III tubulin and TLR4 in the same neurons. Nuclei were stained using DAPI. Data are representative of three independent experiments

Since the receptor for LPS, TLR4, has been reported to be overexpressed in aged brains and AD [10], we investigated next the expression of TLR4 in young (4–8 DIV) and aged cultured (>18 DIV) hippocampal neurons using indirect immunofluorescence followed by optical density analysis. We used this approach instead of the customary western blotting because our hippocampal cultures contain a large fraction of glial cells, about 30% in short-term cultures (3 DIV) that rise to 78–84% in long-term cultures of 10 and 20 DIV, respectively, and also, because of the exceeding sensitivity to trypsin treatment and sorting of aged cells. Figure 2a shows that morphologically identified hippocampal neurons from young and aged cultures express TLR4. Conventional fluorescence images of fixed cells were captured in the same microscopy session using the same lamp and camera settings to ensure comparability. Then, optical density (average fluorescence intensity per area unit) values of selected regions of interest corresponding to morphologically identified neurons were calculated. For this end, fluorescence intensity of the corresponding regions of interest was calculated pixel by pixel, averaged, and divided by the number of pixels for each region of interest (Fig. 2b). The results show that optical density corresponding to TLR4 immunofluorescence increases significantly in aged cultures of rat hippocampal neurons compared to young cultures, consistent with increased expression of TLR4 in the aging brain reported previously in vivo [10]. Twofold immunocytochemistry using antibodies against TLR4 and beta III tubulin confirmed expression of TLR4 in neurons (Fig. 2c). Whereas TLR4 immunofluorescence increases with age in vitro, immunofluorescence of β III tubulin does not change with culture time as reported previously [29]. These results indicate that changes in TLR4 expression are specific and consistent with changes in TLR4 expression reported in vivo*.*

### LPS increases cytosolic [Ca^2+^] in aged cultured hippocampal neurons but not in young cultured neurons

**Fig. 3:**
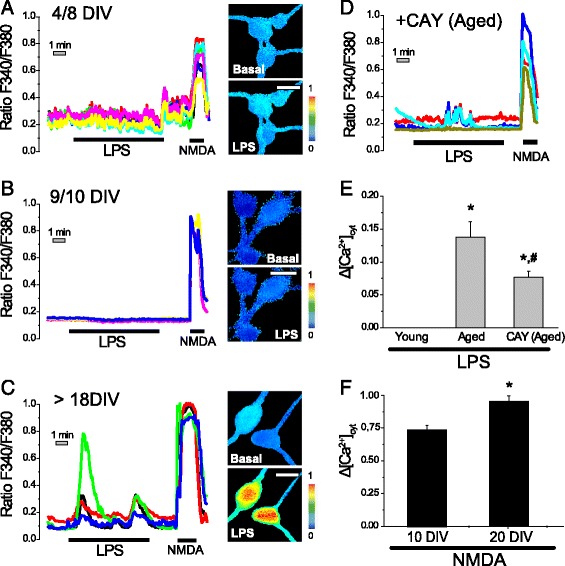
LPS induce Ca^2+^ influx in aged cultures of rat hippocampal neurons but not in young cultures. Hippocampal neurons cultured for 5–8 (young), 9–10 (mature), or >18 (aged and/or senescence) days in vitro (DIV) were loaded with fura2 and subjected to Ca^2+^ imaging. **a**–**c** Traces show representative single-cell [Ca^2+^]_cyt_ responses to 1 μg/ml LPS and NMDA 100 μM added as indicated in 4–8 (**a**), 9–10 (**b**), and >18 DIV (**c**) hippocampal neurons. Pictures at the *right* show pseudocolor images of [Ca^2+^]_cyt_ (Ratio F340/F380) before (basal) and after LPS addition in the same experiments. *Bars* represent 10 μm. **d** Effects of TLR4-activation inhibitor CAY10614 (0.5 μM) on [Ca^2+^]_cyt_ in >18 DIV neurons. **e** *Bars* represent average rises in [Ca^2+^]_cyt_ induced by LPS and calculated as the product of the cell fraction responsive to LPS (1% young = 1% mature, 78% aged, 66% CAY) by the Δ[Ca^2+^]_cyt_ recorded in responsive cells in young neurons, mature neurons, and aged neurons treated with CAY10614 (mean ± SEM; *n* = 45, 24, 23 cells, respectively, from 3–4 independent cultures; *p < 0.05 compared to young; ^#^ *p* < 0.05 compared to aged). **f** *Bars* represent average rises in [Ca^2+^]_cyt_ induced by NMDA in 10 and 20 DIV neurons. *p < 0.05 compared to 10 DIV neurons (mean ± SEM; *n* = 53 and 35 cells, respectively, from 10 and 6 independent cultures)

To ascertain whether TLR4 is functional in rat hippocampal neurons and its activity is modulated by aging and/or senescence in culture, we studied the effect of LPS on [Ca^2+^]_cyt_ in young (4–8 DIV), mature (9–10 DIV), and aged (>18 DIV) cultures of rat hippocampal neurons. For comparison, cells were stimulated also with the glutamate receptor-agonist NMDA that increases [Ca^2+^]_cyt_ specifically in neurons. Cells were loaded with fura2 and subjected to fluorescence Ca^2+^ imaging. Then, the effects of vehicle or LPS (1 μg/ml) on [Ca^2+^]_cyt_ in the absence or the presence of the TLR4-antagonist CAY10614 were tested. Figure 3 shows representative [Ca^2+^]_cyt_ recordings and corresponding Ca^2+^ images coded in pseudocolor of individual neurons identified by morphologic characteristics. We found that both vehicle and LPS have no effect on [Ca^2+^]_cyt_ in 4–8 DIV young (Fig. 3a) or mature 9–10 DIV (Fig. 3b) cultured hippocampal neurons, despite NMDA was able to increase [Ca^2+^]_cyt_ largely in these cells. In contrast, LPS induced clear-cut increases in [Ca^2+^]_cyt_ in most aged neurons studied that were also responsive to NMDA (Fig. 3c). The increase in [Ca^2+^]_cyt_ induced by LPS was partially inhibited by the TLR4-antagonist CAY10614 (Fig. 3d) suggesting that Ca^2+^ responses to LPS in aged and/or senescent neurons depend, at least in part, on TLR4. Average data (Fig. 3e) indicate that Ca^2+^ responses are restricted to aged and/or senescent neurons and prevented by blocking TLR4 activation with CAY10614. In addition, Ca^2+^ responses to LPS are clearly dissociated from Ca^2+^ responses to NMDA that are nearly as large in aged cultured neurons (responsive to LPS) as in mature neurons (unresponsive to LPS) (Fig. 3f).

**Fig. 4:**
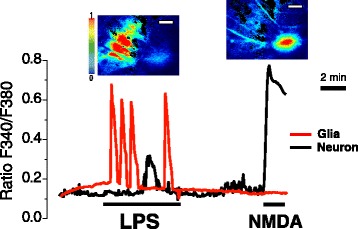
Effects of LPS and NMDA on non-neuronal cells. Hippocampal cells were loaded with fura2 and subjected to Ca^2+^ imaging. Pictures show pseudocolor images of [Ca^2+^]_cyt_ (Ratio F340/F380) after LPS and NMDA in aged hippocampal cells. Traces show representative single-cell [Ca^2+^]_cyt_ responses to 1 μg/ml LPS and NMDA 100 μM in one neuronal cell and one glial cell present in the same microscopic field. Data are representative from eight independent experiments

The effects of LPS in glial cells present in the same hippocampal cultures were also investigated. For this end, Ca^2+^ images in regions of interest corresponding to non-neuronal cells identified morphologically were analyzed. Figure 4 shows representative single-cell recordings of a glia and a neuron from a >18 DIV culture in the same microscopic field challenged sequentially with LPS and NMDA. Only about 30% of the non-neuronal cells displayed Ca^2+^ responses to LPS, often in the form of [Ca^2+^]_cyt_ oscillations. Consistently with a non-neuronal phenotype, these cells were not responsive to NMDA. Ca^2+^ responses to LPS were not observed in non-neuronal cells from young cultures. Interestingly, in some cases in which glial cells and neurons were studied in the same microscopic field (Fig. 4), Ca^2+^ responses to LPS in glial cells preceded Ca^2+^ responses in neurons. However, in other cases Ca^2+^ responses to LPS in neurons occurred in the absence of activation in surrounding glia.

**Fig. 5:**
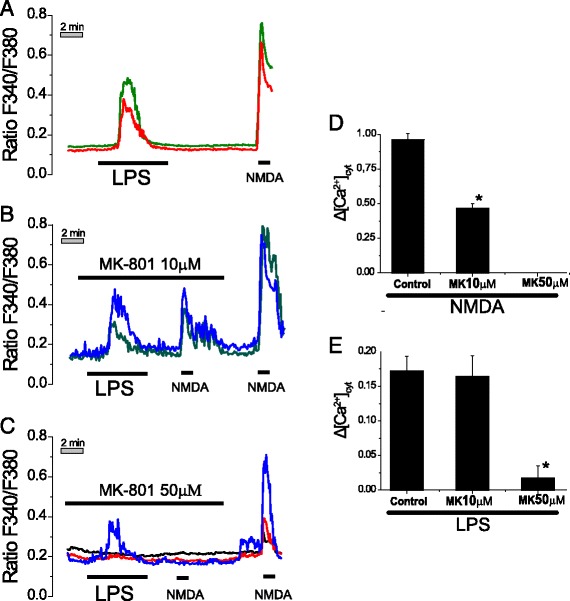
Effects of MK801 on Ca^2+^ responses to LPS and NMDA in aged cultures of rat hippocampal neurons. Hippocampal neurons cultured for >18 DIV were loaded with fura2 and subjected to Ca^2+^ imaging. Traces show representative single-cell [Ca^2+^]_cyt_ responses to 1 μg/ml LPS and NMDA 100 μM in the absence (**a**) and the presence of 10 μM (**b**) or 50 μM (**c**) MK801. *Bars* represent average rises in [Ca^2+^]_cyt_ calculated as in Fig. 3 induced by NMDA (**d**) or LPS (**e**) in the absence (control) and the presence of MK801. Data are from 4–8 independent experiments. Data are from 39 (control), 15 (MK801 10 μM), and 14 (MK801 50 μM) neurons studied in 8, 4, and 5 independent experiments, respectively. *p < 0.05 vs control

The possible contribution of NMDA receptors to Ca^2+^ responses induced by LPS in neurons was also evaluated. For this end we tested the effects of MK801, a well-known NMDA receptor antagonist on Ca^2+^ responses induced by LPS in neurons. Figure 5 shows the effects of LPS and NMDA on [Ca^2+^]_cyt_ in aged hippocampal neurons in the absence (Fig. 5a) and the presence of different concentrations of MK801 (Fig. 5b, c). At 10 μM, MK801 prevented largely the rise in [Ca^2+^]_cyt_ induced by NMDA but had no effect on Ca^2+^ responses induced by LPS (Fig. 5b). However, at 50 μM, a concentration that abolishes the effects of NMDA, MK801 inhibited significantly the Ca^2+^ response induced by LPS (Fig. 5c). Average results are shown in Fig. 5d, e. These results suggest that NMDA receptors are not critical for Ca^2+^ responses to LPS in neurons.

LPS may contribute to cell death in neurons by modulating expression of NMDA receptor subunits in neurons, particularly aged neurons. We have addressed this possibility by testing the effects of LPS treatment (1 μg/m, 24 h) on expression of NMDA receptor subunits in aged neurons using immunofluorescence followed by optical density analysis. Our results indicate that LPS has minor or no effects on expression of NMDA receptor subunits NR1, NR2A, and NR2B in aged neurons (Additional file 1: Figure S1).

### Aβ oligomers enhance LPS-induced Ca^2+^ responses and neuron cell death in aged cultured neurons

**Fig. 6:**
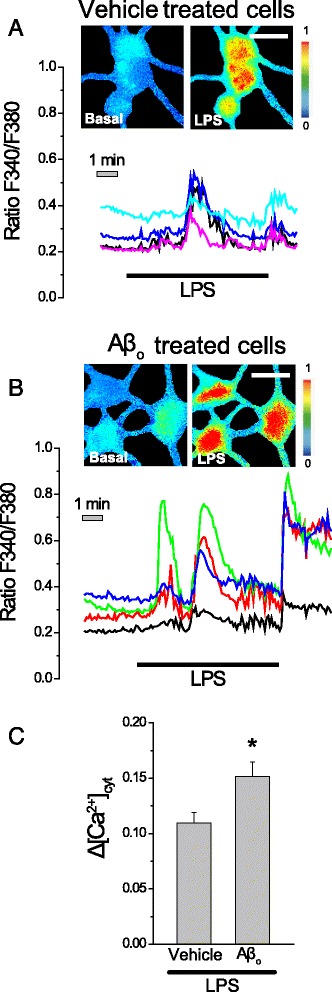
Aβo increase Ca^2+^ responses to LPS in aged cultures of rat hippocampal neurons. Hippocampal neurons cultured for >18 DIV were treated for 48 h with vehicle or Aβo (1 μM), loaded with fura2 and subjected to Ca^2+^ imaging. Pictures show pseudocolor images and representative recordings of [Ca^2+^]_cyt_ (Ratio F340/F380) before (basal) and after LPS addition (LPS) added as indicated in vehicle (**a**) and Aβo treated (**b**) cultured hippocampal neurons. Pseudocolor scales are shown at the *right. Bars* represent 10 μm. **c** *Bars* represent average rises in [Ca^2+^]_cyt_ calculated as the product of the cell fraction responsive to LPS alone (75%) or LPS + Aβo (85%) by the Δ[Ca^2+^]_cyt_ recorded in responsive cells (mean ± SEM; *n* = 62 and 54 cells, respectively, from five independent cultures; *p < 0.05 compared to vehicle)

Aβo are the most like neurotoxin in AD and recent data indicate that they promote hippocampal neuron cell death acting by increasing cytosolic Ca^2+^ [30, 31]. We have asked whether treatment with Aβo may modulate neuron cell death induced by LPS in aged cultures of rat hippocampal neurons. For this end, we assessed the effects of 1 μg/ml LPS on [Ca^2+^]_cyt_ and apoptosis in aged cultures (>18 DIV) of rat hippocampal neurons treated previously with either vehicle or 1 μM Aβo for 48 h. Figure 6 shows representative Ca^2+^ images and effects of LPS on [Ca^2+^]_cyt_ in rat hippocampal neurons treated with vehicle (A) or Aβo (B). We found that 48-h treatment with Aβo increased significantly the rise in [Ca^2+^]_cyt_ induced by LPS in aged cultures of hippocampal cultures (Fig. 6c). These results suggest a cooperation of LPS and Aβo to induce intracellular Ca^2+^ increase in aged cultures of rat hippocampal neurons.

**Fig. 7:**
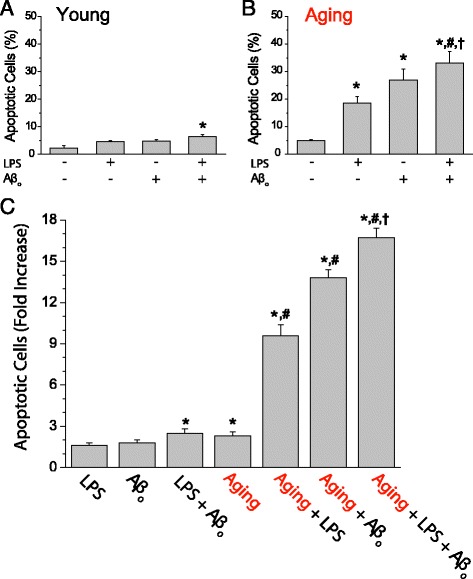
Effect of in vitro aging on LPS and Aβo induced apoptosis in cultured rat hippocampal neurons. Primary hippocampal neurons cultured for 5–8 (young) and >18 (aged) DIV were treated for 48 h with vehicle (control), LPS (1 μg/ml), Aβo (1 μM) or LPS plus Aβo added together. 48 h later, cell apoptosis was assessed by immunofluorescence of Annexin V. Percent of apoptotic neurons in young (**a**) and aged (**b**) cells are shown. Values represent mean ± SEM of three and five experiments from independent cultures, respectively. *p < 0.05 vs. control group; ^#^ *p* < 0.05 vs. LPS group; ^†^ *p* < 0.05 vs. Aβo group. **c** Fold increases in percent of apoptotic cells were calculated from the same data set by dividing the percent of apoptotic cells in each condition by the percent of apoptotic cells observed in young neurons in control conditions. *p < 0.05 vs. LPS group; ^#^ *p* < 0.05 vs. aging alone; ^†^ *p* < 0.05 vs. Aβo group in aging

The effects of LPS on apoptotic cell death in cells previously treated with Aβo were tested next. We found that 48-h exposure to either LPS or Aβo did not promote apoptosis in young cultures of rat hippocampal neurons (Fig. 5a). However, exposure to a combination of both LPS and Aβo induced a small but statistically significant increase in apoptosis in the young cultures of rat hippocampal neurons (Fig. 7a). Interestingly, aged cultures of rat hippocampal neurons were much more sensitive to apoptosis induced by either LPS or Aβo alone (Fig. 7b). In addition, apoptosis further increased significantly when neurons where presented simultaneously with both treatments (Fig. 7b).

To appreciate best the effect of each treatment on apoptosis in the context of aging and/or senescence, we calculated the rise in apoptotic cells (fold increase) induced by each stimuli relative to the percent of apoptotic cells in young cultures treated with vehicle (Fig. 7c). The results show that in young neurons, LPS and Aβo showed a negligible effect on apoptosis. Only when LPS and Aβo were added together, they were able to promote a small, but statistically significant rise in apoptosis, which was similar to the apoptosis observed in untreated aged cultures of neurons (Aging) (Fig. 7c). Remarkably, any of the above treatments added in aged cultures of neurons induced a much larger fold increase in apoptosis, being this effect even significantly larger when both stimuli are present simultaneously in aged neurons. Thus, aging negatively influences hippocampal neuron survival and seems to be a critical factor required for exacerbating LPS- and Aβo-induced neuron death. However, the coincidence of both LPS and Aβo may promote apoptosis also in young neurons, yet to a limited extent.

### Aβo enhance expression of TLR4 in aged cultured neurons

**Fig. 8:**
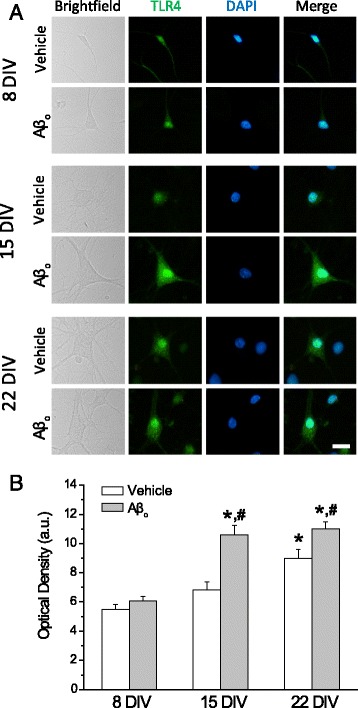
Aβo enhance TLR4 expression in aged cultures of rat hippocampal neurons but not in young cultures. **a** Representative fluorescence images of TLR4 immunofluorescence in 8, 15, and 22 DIV cultured hippocampal neurons. Vehicle refers to images taken from similar cultures in the absence of Aβo treatment. *Scale bar* is 10 μm and applies to all frames. **b** Quantitative analysis of immunofluorescence (Optical Density in arbitrary units) for TLR4. *Bars* represent mean ± SEM (*n* = 25–75 cells from three independent cultures). *p < 0.05 *vs* 8 DIV; ^#^ *p* < 0.05 *vs* vehicle

Next, we addressed whether enhanced susceptibility to LPS induced by Aβo is mediated by TLR4 by testing the effects of Aβo on TLR4 expression. For this end, hippocampal neurons cultured for different DIV were treated for 48 h with vehicle or 1 μM Aβo, and TLR4 expression was tested using immunofluorescence and optical density analysis as in shown in Fig. 2. Figure 8a shows representative bright field and fluorescence images of TLR4 immunostaining (TLR4), nuclei in blue (DAPI) and merge images for hippocampal neurons cultured for 8, 15, and 22 DIV. We found that optical density corresponding to TLR4 immunofluorescence is low in young cultures of neurons as shown above and not modified upon treatment with Aβo (Fig. 8b). At 15 DIV, basal TLR4 immunostaining was still significantly lower than in aged cultures of neurons but it now was significantly increased after Aβo treatment (Fig. 8b). Finally, in aged cultures of neurons (22 DIV), basal TLR4 immunostaining was significantly larger than in 8 and 15 DIV neurons, and further increased with Aβo (Fig. 8b). Thus, the Aβo-promoting effects of TLR4 expression depend strongly on the age of the cultures of neurons, likely reflecting increased susceptibility with aging and/or senescence.

## Discussion

The present study reveals aging and/or senescence as a critical factor required for a marked LPS- and Aβ oligomer-induced neuron cell death as well as for TLR4 expression increase in rat hippocampal neurons. In addition, it discloses an exacerbated interplay between TLR4 and Aβ in the context of aging and/or senescence with consequences in neuronal damage. Our results show that the endotoxin associated to Gram-negative bacteria LPS, by acting on TLR4, increases [Ca^2+^]_cyt_ and promotes cell death in aged cultures of rat hippocampal neurons, but not in young cultures of hippocampal neurons. Several evidences support this conclusion. First, CAY16014, an specific inhibitor of TLR4 activation, inhibits LPS-induced [Ca^2+^]_cyt_ increase and LPS-induced neuron cell death. Second, LPS promotes neuron cell death only in aged cultures of hippocampal neurons that show Ca^2+^ responses to LPS and increased expression of TLR4. In fact, in young cultured neurons expressing low levels of TLR4, LPS did not increase [Ca^2+^] and neither promoted neuronal apoptosis. Thus, aging and/or senescence correlates with enhanced TLR4 expression underlying LPS-mediated, hippocampal neuron death.

We must stress that our results have been obtained in an in vitro model of aging and/or senescence that may not reflect entirely in vivo aging. Evidently, long-term cultured neurons do not undergo the complex processes and interactions involved in in vivo aging. For example, actual young and aged neurons are very different from the metabolic point of view and only limited evidence suggests that these differences remain between short- and long-term cultured hippocampal neurons. However, long-term cultured neurons are amenable for single-cell studies such as the ones performed in this study. In addition, aged cultures of rat hippocampal neurons display many of the hallmarks of aging in vivo, including accumulation of reactive oxygen species, increased oxidative damage of cell proteins, protein carbonyl formation lipofuscin granules, heterochromatic foci, activation of the Jun N-terminal protein kinase and p53/p21 pathways, gradual loss of cholesterol, and changes in Ca^2+^ channel density and NMDA receptor subunits similar to those found in in vivo aging [20, 21]. In addition, Letiembre et al. [10] reported that several TLR mRNAs are upregulated in the mouse-aged brain including TLR4 and its co-receptor CD14, as well as TLR1/2/5/7, while in contrast, transcripts of TLR3/6/8 did not change or even decreased as in the case of *tlr9*. Therefore, long-term cultures of rat hippocampal neurons may provide a reasonable as well as amenable model to investigating aging and/or senescence-related changes in Ca^2+^ signaling in individual neurons. In addition, our results are consistent with increased expression of TLR4 in aged brain reported in vivo [10].

Our results pose the question on whether age-related changes in TLR4 gene expression contribute to susceptibility to neurodegeneration in the elderly. Consistent with this possibility, it has been reported that activation of the central innate immune system may lead to exacerbated neuroinflammation and prolonged sickness behavior in response to LPS in aged mice compared to adult mice [32]. In addition, we have recently reported that increased susceptibility to excitotoxicity and brain damage in aging are also reflected in in vitro aged cultures of rat hippocampal neurons that are much more sensitive to glutamate-induced neuron cell death than young neurons [25]. This effect is induced by dramatic increases in the rise in [Ca^2+^]_cyt_ induced by the glutamate receptor-agonist N-methyl d-aspartate associated to changes in receptor subunits [25] similar to those reported in in vivo aging [20]. Therefore, the aged culture of rat hippocampal neurons may be a good model to assess aging and/or senescence-related changes in the susceptibility to brain damage induced by excitotoxicity and neuroinflammation induced by Ca^2+^ signals that may contribute largely to age-related neurodegeneration. In this regard, it is relevant the mechanism by which LPS increases [Ca^2+^]_cyt_ in hippocampal neurons. In macrophages, it is well established that intracellular Ca^2+^ participates as a second messenger in TLR4-dependent signaling that increases [Ca^2+^]_cyt_ by activating a vanilloid member of the transient receptor potential superfamily of cation channels (TRP channels). In fact, TRPV2 is involved in the LPS-induced Ca^2+^ mobilization from intracellular Ca^2+^ store and extracellular Ca^2+^ [33]. In sensory neurons, however, it has been recently reported that LPS exerts fast, membrane delimited, excitatory actions via TRPA1 [34], another TRP cation channel that is critical for transducing environmental irritant stimuli into nociceptor activity. Surprisingly, the effects of LPS on nociceptors were independent of TLR4 activation [34]. In hippocampal neurons, both LPS-induced [Ca^2+^]_cyt_ rises and neuron cell death were prevented by TLR4 antagonist and the effects correlated with changes in expression of TLR4 suggesting that LPS-induced effects in aged hippocampal neurons depend on TLR4.

We show here that glial cells also show Ca^2+^ responses induced by LPS, sometimes in the form of [Ca^2+^]_cyt_ oscillations preceding Ca^2+^ responses in neurons. These results invite speculation on whether neuronal Ca^2+^ responses to LPS may be secondary to activated glia. For example, TLR4 activation in astrocytes might promote release of glutamate or other molecules that indirectly affect Ca^2+^ currents in neurons. Further research is required to fully exploring this and other possibilities. In any case, direct responses to LPS are definitely involved as most often neuronal Ca^2+^ responses are observed in the absence of glial responses. In addition, TLR4 expression is observed in morphologically and immunologically identified neurons, LPS-induced Ca^2+^ responses in neurons correlated with increased TLR4 expression in aged neurons and are prevented by inhibitors of TLR4 activation.

It could be claimed that LPS effects in aging cultures may be mediated by excitotoxicity events related to changes in NMDA receptor expression. However, several evidences are against this possibility. First, LPS has no or minor effects on expression of NMDA receptor subunits. Second, Ca^2+^ responses to NMDA and LPS are dissociated. Thus, Ca^2+^ responses to LPS are missing in 9–10 DIV neurons that display large Ca^2+^ responses to NMDA. Finally, MK801, a NMDA receptor antagonist, did not inhibit Ca^2+^ responses to LPS at concentrations that inhibit largely Ca^2+^ responses to NMDA.

Neuroinflammation has been associated to neurodegeneration and AD pathology, particularly in the elderly. For example, healthy aged individuals are more likely to suffer profound memory impairments following a challenging life event such as a severe bacterial infection than younger counterparts [35]. Since TLR4 can sense not only molecular patterns derived from bacteria but also from necrotic damage, these data support the hypothesis of a decisive role of aging in exacerbating TLR4-mediated effects. Our data discloses an exacerbated interplay between Aβo and TLR4 in context of aging with consequences in neuronal damage. Specifically, we show here that treatment of rat hippocampal neurons with Aβo increased expression of TLR4, enhanced [Ca^2+^] responses to LPS and increased LPS-induced neuron cell death. Most importantly, all these effects were observed only in aged cultures of rat hippocampal neurons but not in young cultures, indicating that aging and/or senescence is the critical factor required for Aβo-mediated enhancement of LPS-induced damage. It may be argued that coincidence of the neuron damaging effect by LPS and Aβo is very unlikely. However, a non-infectious tissue injury can release TLR4 endogenous ligands called DAMPs that trigger sterile inflammation in the nervous system. Therefore, in the brain, the simultaneous accumulation of Aβ and DAMP-induced TLR4 activation is plausible upon stress and/or brain damage, particularly in the context of aging. In fact, human aging is associated to increased serum levels of pro-inflammatory cytokines, a chronic subclinical condition named as “inflammaging”, as well as increased NF-кB signaling, a transcription factor that is a master regulator of TLR-mediated inflammation. Recent evidences in rat models show that elevation in the levels of inflammatory cytokines induced by TLR4 activation may promote the accumulation of Aβ, which may in turn increase TLR4 levels, thus creating a positive feedback loop that may contribute largely to the progression of AD [36]. This feedback loop may be also amplified further by the age-associated increased expression of NMDA receptors that could be targeted simultaneously by LPS (or DAMPs) and Aβo. Our data is consistent with the report and further demonstrates an exacerbated crosstalk between Aβo and TLR4 on [Ca^2+^] responses and cell death in aged hippocampal neurons, which might be relevant to the pathogenesis of age-related neurodegenerative diseases. Based on this, it is tempting to speculate that aging is the critical contributing factor that enables the vicious cycle between TLR4 and Aβo to promote AD.

## Conclusions

LPS increases cytosolic [Ca^2+^] and promotes apoptosis in rat hippocampal neurons aged in vitro. This effect depends on the age-dependent increase in TLR4 expression that is further enhanced by oligomers of the amyloid β peptide. Thus, TLR4 and amyloid β oligomers cross talk in neuron cell death associated to aging, which might be relevant to AD.

## Additional file

Additional file 1: Figure S1. Effects of LPS treatment on expression of NMDA receptor subunits assessed by immunofluorescence. Representative fluorescence images of NMDA receptor subunits NR1, NR2A and NR2B in aged (>18 DIV) cultured hippocampal neurons. Cells were treated with vehicle or LPS 1 μg/ml for 24 h and then fixed for immunofluorescence followed by optical density analysis. Bars represent values of optical density for NR1, NR2A and NR2B in LPS-treated cells normalized relative to the values obtained in cells treated with vehicle. Data derive from 22, 18, and 38 cells treated with LPS and 21, 37 and 69 cells treated vehicle from 3 independent experiments. **p* < 0.05 *vs* vehicle treated cells. (EPS 2239 kb)

## Abbreviations

[Ca^2+^]_cyt_: Cytosolic-free calcium concentration
AD: Alzheimer’s disease
Aβo: Oligomers of the amyloid β peptide
DIV: Days in vitro
LPS: Lipopolysaccharide
SEM: Standard external medium
TLR4: Toll-like receptor 4
TRP: Transient receptor potential
